# Amalgam restorations for primary molars in clinical practice and dental schools: current situation and required changes

**DOI:** 10.1186/s12903-026-08070-7

**Published:** 2026-03-26

**Authors:** Maha Moussa Azab, Yasmin Mohamed Yousry

**Affiliations:** 1 Faculty of Dentistry, Fayoum University, Fayoum, Egypt; 2 School of Dentistry, Newgiza University NGU, Giza, Egypt; 3 Pediatric Dentistry and Dental Public Health, Faculty of Dentistry, Cairo University, Cairo, Egypt

**Keywords:** Amalgam restoration, Primary molars, Pediatric dentistry courses, Pediatric dentistry curricula

## Abstract

**Background:**

There is a considerable debate in clinical practice regarding the use of amalgam restorations in primary molars. The current study aimed to assess the weight of teaching amalgam cavity preparation and restoration in various pediatric dentistry programs in Egypt, as well as the perceptions of pediatric dentistry educators regarding the teaching and use of dental amalgam as a restoration for primary molars. Additionally, this study sought to correlate the data with the respondents’ demographic data.

**Methods:**

A 24-question web-based questionnaire was created, validated, and sent to heads of pediatric dentistry departments in 30 dental schools in Egypt. The questionnaire was asked to be completed and shared with the teaching staff. The data gathered were exported into an Excel spreadsheet and statistically analysed. Spearman’s rank correlation coefficient and chi-square test for independence were used to assess associations between demographic variables and several statements from the questionnaire.

**Results:**

A total of 139 respondents from 26 schools were included in the data analysis. 21 and 7 schools teach amalgam in undergraduate and postgraduate programs, respectively. 88% of the respondents have not used amalgam for a year or more, 73% will have no actual effect on their practice if amalgam were not an available option. 21% and 42% disagree with continuing to teach amalgam in undergraduate and postgraduate programs, respectively. Statistically significant relations were found between the age, years of experience, type of institution of respondents, and respondents’ agreement with the different statements from the questionnaire concerning the continuation of use and teaching amalgam, as well as increasing the time dedicated to teaching adhesive restorations.

**Conclusions:**

For most paediatric dentistry faculty members, amalgam is not the best choice for restoring primary molars; however, they are cautious and not yet ready to discontinue teaching amalgam, especially among the older and more experienced faculty members. They also believe that the number of training hours dedicated to adhesive restorations should be increased.

**Registration:**

The current study was registered on clinicaltrials.gov on May 28, 2025, with the identifier number NCT06995859.

## Introduction

There is currently a considerable debate in clinical practice about the use of amalgam restorations in primary molars. Following the 2013 Minamata Convention (an international treaty that focuses on combating environmental pollution by hazardous chemicals), the phase-out of dental amalgam was agreed upon due to mercury’s detrimental effects on human health and the environment [1]. Moreover, the European Union committed to combining phasedowns of amalgam and complete phase-outs by 2030 [2]. Despite intense efforts to phase out its use, dental amalgam remains one of the most widely used restorative materials and the foundation of dental services in many countries. This is due to its durability, cost-effectiveness, high strength, and ease of placement [3]. 

The US Food and Drug Administration assessed 34 research studies to provide the most significant and newest information regarding the health risks associated with mercury vapour exposure. Two clinical trials and other retrospective studies found no evidence to support the assertion that mercury exposure through dental amalgams causes unfavourable biological outcomes [4, 5]. The investigation concluded that dental amalgam restorations did not represent a health risk [6]. While some experts declare its practical uses in certain situations and that placing amalgam restorations is crucial for developing hand skills in dentistry, particularly in preclinical settings, justifying its continued teaching relevance in university courses, others propose reducing its emphasis in favour of other alternative restorations [7]. 

In the United Kingdom and Ireland, three surveys were conducted across 18 dental schools, comparing the dental education of composite restorations with that of amalgam restorations. Results showed a decrease in amalgam restorations placed by students, from 70% to 33%, and an increase in composite resin restorations, from 30% to 66%. Despite these educational trends, dental amalgam remains a frequently used material in clinical practice, particularly for posterior restorations, and it remains the preferred material for dental services in many countries, including the United Kingdom [8–10]. 

Although adhesive restorations preserve tooth structure and enhance aesthetic appearance, their durability in primary molars that are subject to high stress or caries risk is limited by their shorter lifespan, technique sensitivity, and increased vulnerability to secondary caries [11]. 

Scientific research, socioeconomic policies, and public opinion all influence the development of the dental curriculum. Dental schools continually review their curricula to ensure that they incorporate modern teaching methodologies and technologies, thereby equipping both graduates and postgraduate students with the necessary knowledge and skills for safe and effective practice [12]. 

While global policies have accelerated the shift away from dental amalgam, the need for national-level evidence is particularly crucial for Egypt, where dental schools play a significant role in shaping clinical practice patterns. To the best of our knowledge, no current data are available regarding the teaching of amalgam restoration in pediatric dentistry at Egyptian universities. Accordingly, the current study aimed to assess the weight of teaching amalgam cavity preparation and restoration in different pediatric dentistry programs, as well as the perceptions of pediatric dentistry educators about teaching and using dental amalgam as a restoration for primary molars, and to correlate this data with the respondents’ demographic data.

## Methodology

### Ethical aspects 

This cross-sectional, descriptive study was registered on ClinicalTrials.gov with the ID NCT06995859. We conducted the survey in accordance with the guiding principles of the Declaration of Helsinki and received ethical approval from the Faculty of Dentistry Research Ethics Committee at Cairo University.

### Drafting the questionnaire

An early version of the questionnaire was developed based on extracted questions from Bissoon and Moodley, as well as Jebur et al. [7, 13], with some modifications and additional questions. The questions were designed to be precise, straightforward, and closely aligned with the study’s objectives.

Five pediatric dental academic experts reviewed the draft questionnaire to assess the content validity and effectiveness of the items addressing the point under investigation. On a 4-point scale, experts evaluated the relevance of each item. The percentage of experts who rated an item as relevant (3 or 4) was used to create item-level content validity indices (I-CVI). Items with I-CVI < 0.78 were revised accordingly. Feedback was considered and utilized to enhance the logical flow, response options, and wording of the questionnaire.

A pilot test for the revised questionnaire was conducted on a subsample of the target population that did not participate in the main study. After completing the questionnaire, participants were asked to rate its comprehensiveness, clarity, and relevance. Finally, adjustments were made to finalize the questionnaire used in this study.

The final questionnaire consisted of closed-ended questions (such as multiple-choice and Likert scales). It was divided into three sections: The first section collected demographic information from the participants (5 items). The second section included questions addressing the weight of “amalgam cavity preparation” teaching and training in undergraduate and postgraduate curricula (10 items). The third section assessed participants’ practices and perceptions regarding the teaching and use of amalgam in restoring primary molars (9 items).

### Inclusion and exclusion criteria for participants

Pediatric dentistry educators from Egyptian universities with at least one year of experience were included in the study. However, pediatric dentistry educators who were unwilling to participate or complete the questionnaire, as well as administrative and non-teaching staff, were excluded from the study.

### Data collection

A web-based questionnaire was created and hosted on a secure online survey platform [Google Forms], which was chosen for its data security features and compatibility with digital devices.

Heads of pediatric dentistry departments of 30 dental schools in Egypt were contacted via email to introduce the study and request their staff’s participation in the survey. Each email contained a direct link to the questionnaire, a brief explanation of the study’s objectives, an estimated completion time, and a statement regarding confidentiality and voluntary participation. The heads of departments were asked to share the link with all department teaching staff and encourage their participation. The study was also promoted to increase participation and involvement through internal communication channels, including the university’s staff portal and academic staff groups on social media.

By completing the questionnaire, participants consented to take part, and they were free to withdraw by not completing it. The response time frame was 3 to 4 weeks. We sent two reminder emails to encourage participation, one after 2 weeks and another just before the deadline. Finally, data gathered from questionnaire responses were exported into an Excel spreadsheet and statistically analysed.

### Statistical analysis

Statistical analysis was performed using Microsoft Excel software for Windows. Categorical data were presented as frequency and percentage values. Spearman’s rank correlation coefficient was used to assess correlations between ordinal variables and the respondents’ agreement level to several statements from the questionnaire, while the chi-square test for independence was used to test the associations between nominal variables and the respondents’ agreement level to the same statements from the questionnaire. For all tests, the significance level was set at *p* < 0.05.

## Results

One hundred ninety-six respondents filled out the sent questionnaire. However, 54 responses were excluded because the respondents did not work in educational institutions, and three additional responses were excluded because they worked at foreign universities, leaving 139 responses to be included in the data analysis. The demographic data for the respondents is presented in Table 1.

**Table 1:** Summary of participants' demographic data

Background characteristics	Variable	N	%
Gender	Women	102	78.4%
	Men	37	21.6%
Practice sector	Educational Institutions	33	23.7%
	Educational institutions + clinical practices	106	76.3%
Institution	Governmental University	87	62.6%
	Private University	53	37.4%
Age	20-30	63	45.3%
	31-40	47	33.8%
	41-50	20	14.4%
	More than 50	9	6.5%
Years of experience	1-5	62	44.6%
	6-10	27	19.4%
	11-15	22	15.8%
	16-20	12	8.6%
	More than 20	16	11.5%

For the second part of the questionnaire, which assessed the current situation in different universities in Egypt, results were analysed so that each university was considered as a single respondent. Universities’ results were generated by summarizing the responses of each university’s participants, for each item, using the (mode) function, which represents the response that was most frequently chosen. This approach provides a more accurate reflection of each dental school than individual responses, and it helps prevent the bias that could occur if one institution contributed more responses than others, assuming that responses regarding teaching and clinical practice within the same university would be broadly consistent. The responses were from 26 different universities and are presented in Table 2.

**Table 2:** Distribution of responses across universities (calculated separately for each university based on participants' responses) concerning amalgam cavity preparation teaching and training

Questions	Answers	N	%
In the pediatric dentistry undergraduate curriculum, do you theoretically teach amalgam cavity preparation for primary molars? (N=26)	Yes	20	77%
	No	6	23%
How many theoretical hours (lectures or small groups) are dedicated to amalgam? (N=20)	1 hour	11	55%
	2 hours	5	25%
	3 hours	1	5%
	4 hours	2	10%
	5 or more hours	1	5%
Do you teach amalgam cavity preparation in primary molars in pediatric dentistry undergraduate simulation labs/clinics? (N=26)	Yes	21	80.%7
	No	5	19.3%
How many hours in labs or clinics are dedicated to amalgam? (N=21)	1-5 hours	10	47.6%
	6-10 hours	6	28.6%
	11-15 hours	3	14.3%
	16-20 hours	1	4.8%
	more than 20 hours	1	4.8%
Do interns practice amalgam cavity preparation and restoration for primary molars? (N=26)	Yes	10	38.5%
	No	16	61.5%
Do you have a postgraduate pediatric dentistry program? (N=26)	Yes	12	46.2%
	No	14	53.8%
In the pediatric dentistry postgraduate curriculum, do you theoretically teach amalgam cavity preparation and restoration for primary molars? (N=12)	Yes	7	58.3%
	No	5	41.6%
How many theoretical hours (lectures or seminars) are dedicated to amalgam cavity preparation and restoration -for primary molars- in the postgraduate pediatric dentistry program? (N=7)	1 hour	2	28.6%
	2hours	4	57.1%
	3 hours	1	14.3%
Do the postgraduate students practice amalgam cavity preparation and restoration for primary molars? (N=7)	Yes	4	57.1%
	No	3	42.9%
How many amalgam cavity preparation and restoration cases must each postgraduate student perform? (N=4)	1-5 cases	1	25%
	6-10 cases	1	25%
	11-15 cases	2	50%

The third part of the questionnaire assessed the practice and insight of the respondents (*N* = 139) regarding the use, teaching, and future of amalgam restorations, presented in Table 3.

**Table 3:** The distribution of participants’ responses concerning the teaching and use of amalgam in restoring primary molars

Questions	Answers	N	%
How often do you apply amalgam restoration for primary molars in your clinical practice?	Always	1	0.7%
	Often	7	5%
	Sometimes	29	20.9%
	Rarely	38	27.3%
	Never	62	44.6%
When did you last apply an amalgam restoration to a primary molar?	In one week	17	12.2%
	Last year	19	13.7%
	In 2 years	12	8.6%
	In more than 2 years,	47	33.8%
	Did not apply amalgam at all.	44	31.7%
If you were not able to use amalgam as a restorative material, how would this affect your pediatric dentistry practice?	No effect	102	73.4%
	Medium effect	32	23%
	Huge effect	5	3.6%
“Amalgam is still a useful option for restoring primary molars.”	Agree	36	25.9%
	Neutral	38	27.3%
	Disagreed	65	46.8%
Regarding Amalgam cavity preparation and restoration in primary molars, there is a real difference, a ‘disconnect’, between what is taught at university and what is experienced in real life.	Agree	53	38.4%
	Neutral	59	42.8%
	Disagreed	26	18.8%
"We should continue teaching amalgam cavity preparation and restoration for primary molars for undergraduate students."	Agree	40	28.8%
	Agree with reducing the time of teaching amalgam	58	41.7%
	Neutral	11	7.9%
	Disagree	30	21.6%
"We should continue teaching amalgam cavity preparation and restoration for primary molars for postgraduate students."	Agree	26	18.7%
	Agree with reducing the time of teaching amalgam	36	25.9%
	Neutral	18	12.9%
	Disagree	59	42.4%
"We should invest more time in postgraduate education and training (continuing professional development) in using adhesive restorative materials."	Agree	117	84.2%
	Neutral	15	10.8%
	Disagreed	7	5%
"We should phase out the use of amalgam in pediatric dental practice in Egypt."	Agree	64	46%
	Neutral	56	40.3%
	Disagreed	19	13.7%

**Fig. 1:**
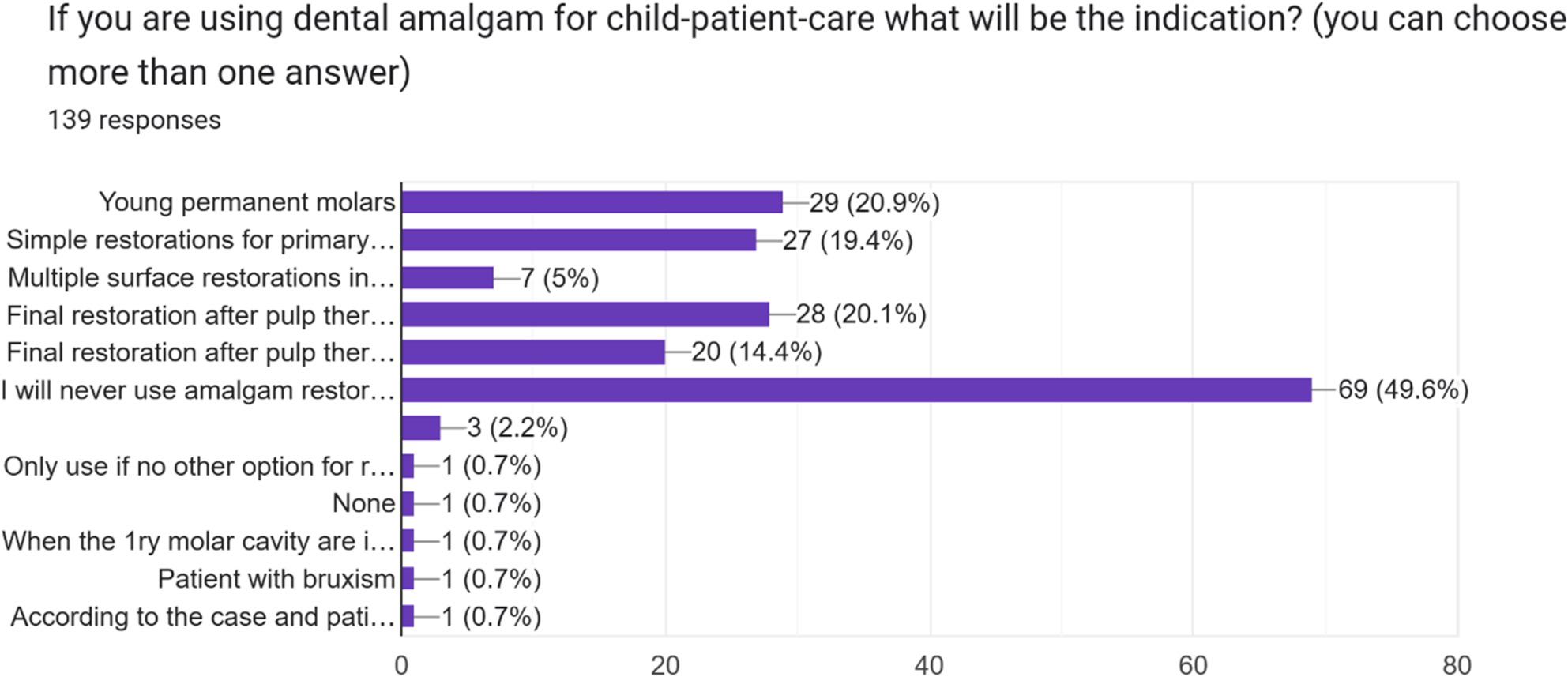
Participants' responses to the question concerning the indication of use of amalgam

**Fig. 2:**
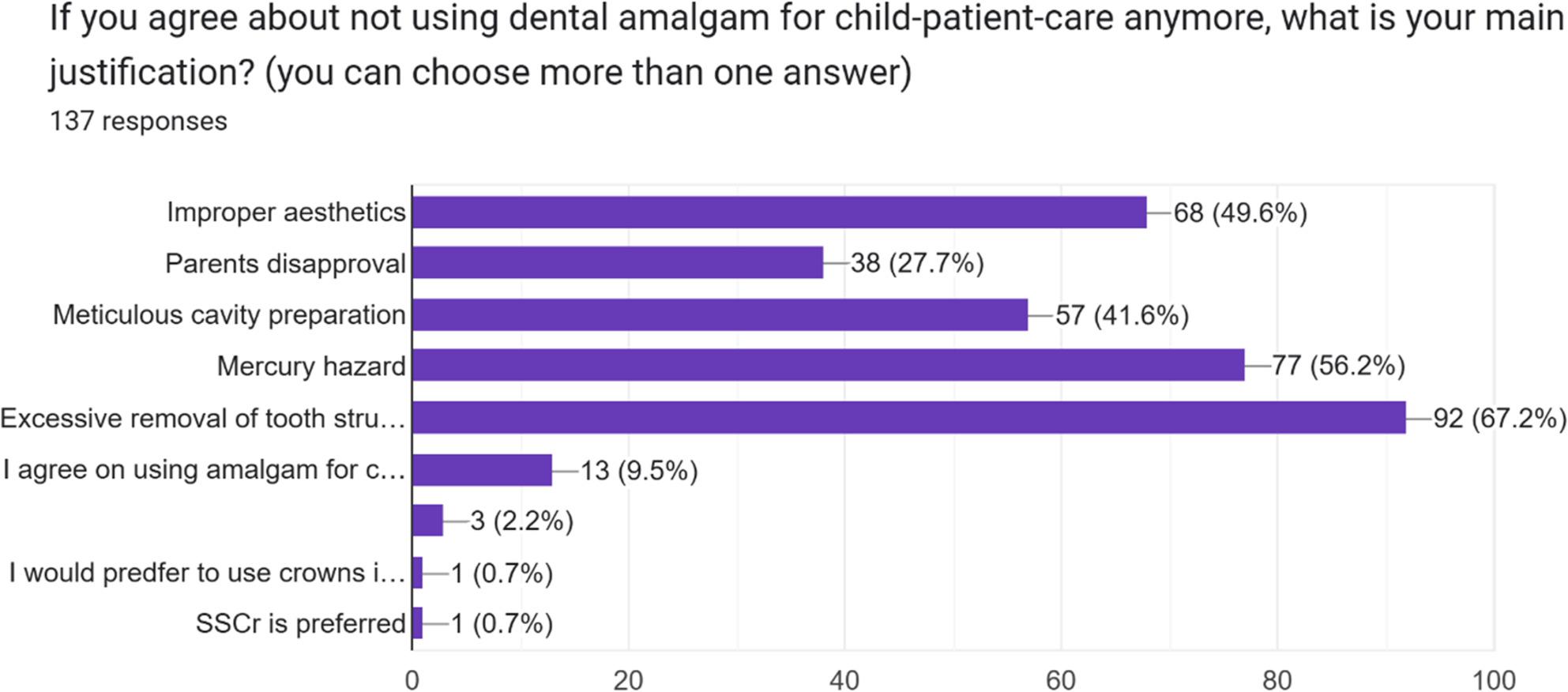
Participants' responses to the question concerning the justification for not using amalgam

According to the respondents, “the indications for using amalgam restorations for a child patient” and “the justification for not using them” are presented in Figs. 1 and 2

Statistically significant weak to moderate positive correlations were found between the age of the respondents and statements (Amalgam is still a useful option for restoring primary molars), (We should continue teaching amalgam cavity preparation and restoration for primary molars for undergraduate students), and (We should invest more time in postgraduate education and training in using adhesive restorative materials). Additionally, a statistically significant moderate positive correlation was found between the respondents’ years of experience and the statement (We should invest more time in postgraduate education and training in using adhesive restorative materials). Additionally, a weak statistically significant association was found between the type of institution of respondents and their agreement with the statement (We should continue teaching amalgam cavity preparation and restoration for primary molars to undergraduate students). The results are presented in Table 4.

**Table 4:** The association between participants' responses with respect to the teaching and use of amalgam in the restoration of primary molars and respondents’ age, years of experience, type of institution, and practice

Items	Age	Years of experience	Type of institution	Practice sector
Amalgam is still a useful option for restoring primary molars.	0.279p=0.00064*	–0.029p=0.735.	3.82p=0.15	2.27 p=0.32
We should continue teaching amalgam cavity preparation and restoration for primary molars for undergraduate students.	0.308p=0.00014*	–0.062p=0.467	7.35 p=0.025*	2.28 p=0.32
We should continue teaching amalgam cavity preparation and restoration for primary molars for postgraduate students.	-0.05p=0.561	0.018p=0.836	2.34p=0.31	0.16p=0.92
We should invest more time in postgraduate education and training (continuing professional development) in using adhesive restorative materials.	0.35p=0.00001*	0.446p≈0*	5.58p=0.06	2.94​p=0.23
We should phase out the use of amalgam in pediatric dental practice in Egypt."	0.08p=0.346	0.145p=0.085.	1.77p= 0.41	0.77p=0.68

*; significant (*p*<0.05)

## Discussion

Dental education has traditionally focused on developing the clinical skills necessary for restorative procedures. Amalgam restorations have been a cornerstone of the dental curriculum for many years. However, due to environmental concerns about mercury content and the growing popularity of composite materials, the use of amalgam has become a subject of debate in recent years [1]. Thus, this study aimed to assess the current situation of teaching amalgam cavity preparation and restoration in Egyptian universities, and to have an overview of how pediatric dentistry educators use amalgam and what they consider the best approach to teaching amalgam in pediatric dentistry courses [14]. 

In this study, the higher response rate among junior and female educators may be attributed to the strong motivation and frequent participation in academic career progress, networking, and exposure that younger educators experience during their early professional stages. Similarly, females may be more interactive due to their drive to promote mentorship and their sense of administrative responsibility [15, 16]. 

For the second part of the questionnaire, which deals with the current situation in various dental institutions in Egypt, it seems normal that about 80% of dental schools still teach amalgam for only one or a few hours. In most Egyptian dental schools, pediatric dentistry courses are preceded by conservative dentistry courses in earlier years, which may explain why schools that teach amalgam in pediatric dentistry do it in a course of limited hours. Some schools do not teach amalgam in their pediatric dentistry courses and instead rely on conservative dentistry departments to do so.

Through the postgraduate program, various courses are taught, with dedicated courses focused on the conservative restoration of primary teeth. Despite the fact that amalgam is primarily taught in detail in the conservative dentistry specialty, one cannot disregard the necessity for a pediatric dentist to understand the differences between applying amalgam in a permanent molar and a primary molar. Also, a previous study by Bhayat et al. [17] threw light on the problem of the requested quota for clinical requirements in dental programs, where students are obliged to do a certain number of specific restorations to fulfill their requirements, even when the students think it is not their preferred restoration in general or for a particular case. They recommended reducing the amalgam clinical quota and that clinical requirements be designed so that the choice of restorative material depends solely on the clinical situation, rather than on a pre-decided quota.

The third part of the questionnaire assesses the respondents’ practices and perceptions regarding the use and teaching of amalgam restorations. Most respondents rarely or never apply amalgam restorations for primary molars in their clinical practice. This aligns with the work of Dentino et al. [18], who found that the number of amalgam restorations applied is generally in decline, and pediatric dentists are less likely to use amalgam than general dentists.

For the question about “the effect of not using amalgam on their clinical practice,” the majority (73.4%) responded that there would be no effect. This does not agree with a previous study by Jebur et al., [7], where the general dentists perceived the absence of the amalgam restoration option as having a significant impact on their practice, specifically for cases with difficult moisture control and subgingival restorations, in the same way, 46.8% of respondents do not think that “Amalgam is a useful option for restoring primary molars anymore,” which disagrees with a previous study that found that 74% of the responding American schools used amalgam in primary posterior teeth in their predoctoral pediatric dentistry clinics [19]. These variations in results could be attributed to differences in geographic regions and types of practices, where dentists in educational dental clinics often recommend amalgam to their patients, who are typically at high risk for caries and usually lack medical insurance coverage [19, 20]. 

Approximately 70% of respondents believe that the undergraduate curriculum of pediatric dentistry should still include teaching amalgam, while only 45% agree that it should continue to include amalgam in postgraduate curricula. This comes surprisingly at first, since it came in contrast to the answers of previous questions like “do you think amalgam is a useful option for restoring primary molars” and “effect of not using amalgam on your practice”; however, about 2/3 of those who agree on continuing to teach amalgam think we should decrease the number of teaching hours. Several previous studies have shown a reduction in the hours of teaching amalgam in dental schools in general [7, 21]. Also, American dental universities did not agree with phasing out amalgam teaching in their curricula [19]. 

For many educators, the GV Blacks principles for cavity preparation, which were primarily designed for receiving amalgam restorations, are the cornerstone for studying and understanding conservative dentistry. Usually, all that follows is in the form of modifications and alterations to these principles. Additionally, the meticulous design of a cavity to receive amalgam is a valuable educational practice that enables students to master the use of dental armamentarium and enhance their manual dexterity, even when practiced only in simulation labs [22]. 

The recommendation to reduce amalgam teaching time aligns with the fact that 84.2% of respondents support increasing the time spent teaching and practicing adhesive restorations in postgraduate pediatric dentistry programs. This aligns with the aforementioned situation in the dental practices of the respondents, where most of them rarely place amalgam and primarily depend on adhesive restorations. A Previous study found an increase in hours dedicated to teaching adhesive restorations in comparison to amalgam. This study also highlights the fact that the increase in the number of dental students, with limited facilities and available study hours, makes it crucial to wisely dedicate the available time, facilities, and teaching manpower to what will better serve the dental students and their future patients [13]. 

As a summary of the previous questionnaire and in relation to the current situation, 46% of respondents agreed with the statement “phasing out the use of amalgam in pediatric dental practice in Egypt.” In Bissoon et al.‘s study [13], 25% of the faculty thought that amalgam should be totally dismissed from clinical use. In contrast, all clinicians believed that amalgam should remain a restorative option. On the other hand, Aggarwal et al. [21] considered the phase-out of amalgam a step that could increase inequalities in delivering dental services until less expensive, long-lasting restorable materials are available. 

The results of this study indicate that older respondents with more years of experience are more likely to agree with statements concerning the continuation of using and teaching amalgam, as well as increasing the time dedicated to teaching adhesive restorations in pediatric dental postgraduate studies. This finding aligns with previous research suggesting that senior dentists—those 40 years or older—are more likely than younger dentists to employ and support amalgam restorations. This may be connected to their clinical experience with amalgam’s durability and reliability, as well as their training period, when it was the most common restorative material. Amalgam usage may be more familiar and comfortable to older generations, whereas younger generations may be more ecologically conscious and concerned about health hazards, preferring alternatives [23, 24]. 

Also, a weak statistical significance association showed that respondents from governmental institutions have slight tendencies towards disagreement with (the teaching of amalgam for undergraduate students). This finding was in contrast to previous studies [19, 25], which revealed that private schools tend to disagree more about continuing amalgam teaching, justifying this stance by having more aesthetic restoration-focused patient pools and modern facilities, which can influence educators’ attitudes.

This study has some limitations, due to the absence of data regarding the accurate count of faculty members in each university, it was not feasible to calculate a sample size or response rate. The vast difference in the number of faculty members in different universities may lead to some bias, as the choices of faculty members from universities with a larger number of participating faculty will largely influence the total responses to the questions addressing the current status of teaching amalgam in these universities. We tried to overcome this limitation by considering the choices of respondents from the same universities as one count, to avoid the overwhelming effect of responses from universities with a larger number of participants. Additionally, it should be acknowledged that not all dental schools were reachable, and we didn’t receive responses from some dental schools. Consequently, the collected data may not fully represent the perceptions of all dental educators in Egyptian dental schools. However, the responses obtained could provide valuable insights into the overall situation. Further studies, including those involving more universities across different regions and utilising mixed approaches that combine surveys with interviews, could provide a more comprehensive picture to develop consensus-based recommendations for improving dental curricula, continuous training, clinical practices, and policies in Egypt.

## Conclusion

The results of this study show some variation in the use of restorative materials for primary molars; however, amalgam is not the preferred restorative choice for most pediatric educators. In general, most paediatric dentistry faculty members, especially the older and more experienced, are cautious about discontinuing the teaching of amalgam in paediatric dentistry courses, but they suggest decreasing the number of teaching and training hours and dedicating them to other alternatives, such as adhesive restorations. Assessing current teaching practices and educators’ perspectives in Egypt is crucial for planning practical and sustainable curricular reforms that enhance dental students’ knowledge and practical skills, which will directly influence their confidence, material selection, and adherence to future national guidelines. Additionally, this helps highlight areas where training can be enhanced to align with international trends, supporting Egypt’s efforts toward environmentally conscious dental practices.

## Data Availability

All data generated or analysed during the study are included in this research article.
